# Distribution of Airborne SARS-CoV-2 and possible aerosol transmission in Wuhan hospitals, China

**DOI:** 10.1093/nsr/nwaa250

**Published:** 2020-09-28

**Authors:** Jia Hu, Chengfeng Lei, Zhen Chen, Weihua Liu, Xujuan Hu, Rongjuan Pei, Zhengyuan Su, Fei Deng, Yu Huang, Xiulian Sun, Junji Cao, Wuxiang Guan

**Affiliations:** Wuhan Institute of Virology, Center for Biosafety Mega-Science, Chinese Academy of Sciences, China; Wuhan Institute of Virology, Center for Biosafety Mega-Science, Chinese Academy of Sciences, China; Wuhan Institute of Virology, Center for Biosafety Mega-Science, Chinese Academy of Sciences, China; Wuhan Jinyintan Hospital, China; Wuhan Jinyintan Hospital, China; Wuhan Institute of Virology, Center for Biosafety Mega-Science, Chinese Academy of Sciences, China; Wuhan Institute of Virology, Center for Biosafety Mega-Science, Chinese Academy of Sciences, China; Wuhan Institute of Virology, Center for Biosafety Mega-Science, Chinese Academy of Sciences, China; Key Lab of Aerosol Chemistry and Physics, SKLLQG, Institute of Earth Environment, Chinese Academy of Sciences, China; Wuhan Institute of Virology, Center for Biosafety Mega-Science, Chinese Academy of Sciences, China; Key Lab of Aerosol Chemistry and Physics, SKLLQG, Institute of Earth Environment, Chinese Academy of Sciences, China; Wuhan Institute of Virology, Center for Biosafety Mega-Science, Chinese Academy of Sciences, China

## Abstract

SARS-CoV-2 viral RNA was found in 21% of the aerosol (n = 38) and 39% of the mask (n = 23) samples collected from Wuhan hospitals early in 2020. Viable virus was isolated from one surgical mask, but not from any of the viral RNA positive aerosol samples.

The novel severe acute respiratory syndrome coronavirus 2 (SARS-CoV-2) has caused an outbreak of COVID-19 (2019 coronavirus infectious disease) that has led to a global public health crisis. The spread of SARS-CoV-2 can be rapid, but there is still considerable controversy about its main route of transmission (1,2); that is, whether transmission is by contact or through the air (2,3). Recently WHO (World Health Organization) released a document that recognized the possibility of aerosol transmission of SARS-CoV-2 for indoor environments and enclosed spaces (4). The purpose of the present study was to evaluate the airborne transmission of SARS-CoV-2 through a field investigation of its occurrence in the air and other samples collected from health facilities in Wuhan, China.

ORIGINAL UNEDITED MANUSCRIPT

To characterize the distribution of SARS-CoV-2 in selected microenvironments, 123 indoor and outdoor air samples were collected from various locations in the Jinyintan, Hongshan Square Cabin, and Union hospitals in Wuhan, China from 16 February to 14 March 2020. The locations of the sampling sites and descriptions of the microenvironments where the samples were taken are summarized in Figures S1 and Table S1). We used a qRT-PCR (quantitative Reverse Transcription real-time fluorescence Polymerase Chain Reaction) kit targeting the *Orf1ab* gene to quantify the genome copy numbers of the SARS-CoV-2 (Materials and Methods are in the Supplementary Material). Eight of 38 aerosol samples (21.1%) from intensive care units (ICUs) and one of six aerosol samples (16.7%) from Computerized Tomography (CT) rooms were positive for viral RNA (Figure 1, Table S1). The range of virus concentrations in the positive aerosol samples was $1.11 \times 10^3$ to $1.12 \times 10^4$ RNA copies $m^{-3}$. The relatively high virus RNA detection rates in the ICU ward and CT room where patients' activities were concentrated are consistent with a previous study (5). The aerosol samples from other areas of the hospitals, including medical staff rest areas and corridors, were all negative, which may have been due to good ventilation and clean air in those areas. We found positive viral RNA in 20% and 10% of the outdoor air samples collected 10-m from the doors of inpatient and outpatient buildings, respectively (Figure S1), with concentrations ranging from 0.89-$1.65 \times 10^3$ RNA copies $m^{-3}$. All viral RNA positive aerosol samples were subjected to cell culture to determine whether viable virus could be recovered from them. The viral nucleic acid tests were negative after three passages of Vero-E6 cells inoculated in a blind test. Similarly, no viable virus was isolated from SARS-CoV-2 PCR-positive air samples in a study conducted at Nebraska, USA (3).

To determine whether surgical masks helped prevent patients from spreading SARS-CoV-2 in exhaled air, we collected 23 surgical masks from patients who had a range of health conditions, from critically to severely to mildly ill, and then tested the masks for the presence of viral RNA (Figure S2). The qRT-PCR results showed that 39.1% (9 of 23) of the masks were positive, and positive detection rates ranged from 30% to 50% for the three types of patients (Fig. S2A). Viral RNA varied from

ORIGINAL UNEDITED MANUSCRIPT

$5.71 \times 10^3$ to $1.90 \times 10^7$ copies/mask (Fig. S2B). These results indicate that exhaled breath can be an important source for SARS-CoV-2, and the positive rates for the mask samples were relatively high (Fig. S2) compared with the aerosol (Fig. 1) or surface samples (Fig. S3). All positive masks were subjected to cell culture and inoculated with Vero-E6 cells after blind passage for three generations. One mask from a critically ill patient detected positive, suggesting that the mask did indeed block the release of viable virus in the air exhaled from the patient.

To evaluate the role of masks in preventing the spread of viruses to healthy persons, we tested 10 filters from respirators and 40 masks from healthy researchers working in the P3 laboratory. All respirator filter samples were positive for SARS-CoV-2 RNA while the masks, which were positioned downstream of the respirators, were all negative, indicating that the respirator filters effectively blocked the transmission of virus through the air. Indeed, the negative results for the masks suggest that the respirators provide a high level of filtration efficiency and protection for airborne virus particles and that the air inhaled by the researchers who wore them was not contaminated with virus.

We also collected environmental samples near COVID-19 patients in the hospitals, and of these, five surface swabs (cabinet, patient's bedrail, door handle, and patient monitor) out of 24 (20.8%) from the ICU were positive for SARS-CoV-2, with viral RNA (Fig. S3A) ranging from $1.52 \times 10^3$ to $4.49 \times 10^3$ copies/swab (Fig. S3B). Our results show that efficient disinfection is critical for nosocomial infection control and protection of medical staff. Positive virus RNA was found for surface samples from a cabinet (2 of 2, 100%), an ill patient's bedrail (1 of 2, 50%), a door handle (1 of 2, 50%) and a patient monitor (1 of 2, 50%). However, after rigorous disinfection, no viral RNA was detected in a second batch samples from the same places.

In our study, the SARS-CoV-2 RNA positive masks from patients (exhaled from infected patient), ambient air (transmission medium), and respirators (inhaled by healthy persons) compose a transmission chain from emission to transport to acquisition of the virus, and this is indirect evidence that, at least under some

ORIGINAL UNEDITED MANUSCRIPT

circumstances, SARS-CoV-2 may spread through the air. It is noteworthy that aerosol transmission of SARS-CoV-2 has been reported in several cases involving family gatherings in various Chinese cities (6,7), a restaurant in Guangzhou, China (8), and a choir rehearsal in the USA (9). Sia et al. demonstrated that SARS-CoV-2 transmitted efficiently from inoculated hamsters to naive hamsters via aerosols (10).

Our results indicate that masks can play an important role in preventing patients from exhaling virus particles and reducing the chances that healthy people will be infected. Therefore, the wearing of masks should be encouraged for preventing the spread of COVID-19, and other measures, such as improvements to ventilation and air disinfection, also should be adopted for enclosed and semi-enclosed spaces.

**Author Contributions**

W.X.G. and J.J.C. designed the study. C.F.L., J.H., W.H.L., X.L.S., W.X.G., F.D, Z.Y.S., Z.C., R.J.P. collected and analyzed the samples. W.X.G., J.J.C., J.H., F.D. interpreted the data. W.X.G., J.J.C. and X.L.S. plotted the figures and wrote the manuscript with contributions from co-authors.

**ACKNOWLEDGMENTS**

We thank Dr. Jianming Qiu (Kansas University of Medical Center, Kansas City, Kansas, USA) for critical suggestions.

**FUNDING**

We appreciate the support from the Ministry of Science and Technology (2020YFC0845800, 2020YFC0842500), Chinese Academy of Sciences (2020YJFK0107), Chinese Academy of Engineering (2020-ZD-15) and the National Natural Science Foundation of China (52041006).

We declare no competing interests.

ORIGINAL UNEDITED MANUSCRIPT

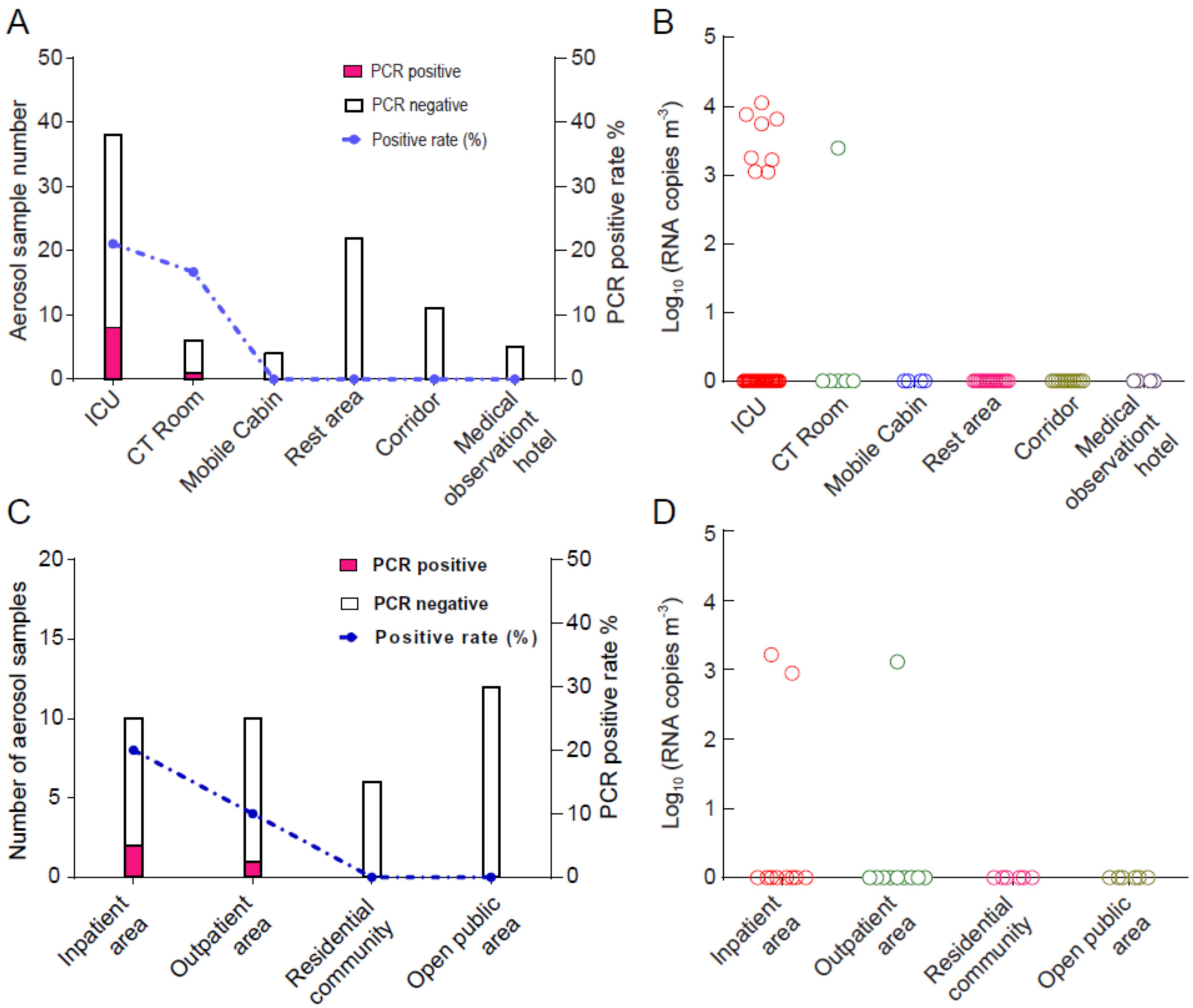

**Figure 1. Detection of SARS-CoV-2 in the aerosol samples in Wuhan hospitals, China.**

Aerosol samples from various locations were collected over 30 min with centrifugal bioaerosol samplers. qRT-PCR targeting the *Orf1ab* gene of the SARS-CoV-2 was performed using One Step PrimeScript™ RT-PCR kits.

A and C: The numbers of qRT-PCR positive and negative aerosol samples from (A) indoors or (C) outdoors are shown as filled and open bars, respectively; the dashed line represents the qRT-PCR positive rates of the aerosol samples;

ORIGINAL UNEDITED

B and D: Copy numbers of SARS-CoV-2 RNA in aerosols from (B) indoors or (D) outdoors. A standard curve ($Y = 47.951 - 3.44 \times \log$ (copy numbers)) generated from the *in vitro* transcribed RNA was used to calculate the copy numbers of SARS-CoV-2 RNA.

ORIGINAL UNEDITED MANUSCRIPT
