# Supplementary material for: Distribution of airborne SARS-CoV-2 and possible aerosol transmission in Wuhan hospitals, China
Source: Natl Sci Rev. 2020 Sep 28;7(12):1865–7. doi: 10.1093/nsr/nwaa250 (PMC7543474; doi:10.1093/nsr/nwaa250)
Supplement: nwaa250_Supplemental_File [file nwaa250_supplemental_file.doc]

**Materials and Methods**

**Sampling**

Aerosol samples were collected over 30 min intervals from February 16 to March 14, 2020 with the use of a centrifugal aerosol-to-hydrosol sampler (WA-400, Beijing Dingblue Technology Co., Ltd., China). The sampler had a 50% aerodynamic equivalent cut-off diameter of 0.8 μm and operated at a flow rate of 400 L/min (a total air volume of 12 m^-3^ was sampled). The aerosols were removed from the sampler with 2 ml of phosphate buffered saline (PBS) containing antibiotic-antimycotic (Life Technologies Co., NY, USA) and 0.5% bovine serum albumin (BSA) (Amersco, Ohio, USA). A total of 81 aerosol samples were taken from selected locations in the Wuhan Jinyintan Hospital, Hongshan Square Mobile Hospital and Union Hospital, Tongji Medical College, and Huazhong University of Sciences and Technology. An additional 42 aerosol samples were collected in medical observation hotels, residential communities, gardens, and greenways. Twenty-three masks from patients and 24 swabs from surfaces in ICUs were also collected and analyzed. Ten 3M™ Versaflo™ TR-600 respirator filters (3M, Minnesota, USA) and 40 masks from healthy workers in the P3 lab of Wuhan Institute of Virology were collected for viral RNA detection. The airflow rate of the respiratory filters was 190 L/min and the surface area was ~30 cm^2^.

**RNA extraction**

The swabs and patient masks were washed six times with 1 ml PBS, and 1 ml of TRIzol™ Reagent (Invitrogen, NY, USA) was added to 1 ml of the aerosol washings. The viral RNA was isolated following a standard protocol according to the manufacturer’s instructions and dissolved in 20 μl of diethyl pyrocarbonate (DEPC)-treated water.

**Quantitative Reverse Transcription Polymerase Chain Reaction (qRT-PCR)**

qRT-PCR analyses were performed using One Step PrimeScriptTM RT-PCR kits (Perfect Real Time, Takara, RR064A), following the standard protocol provided by the manufacturer. The *Orf1ab* primers (*Orf1ab*-F: 5’-CCC TGT GGG TTT TAC ACT TAA-3’, *Orf1ab*-R: 5’-ACG ATT GTG CAT CAG CTG A-3’) and probe (*Orf1ab*-P 5'-FAM-CCG TCT GCG GTA TGT GGA AAG GTT ATG G-BHQ1-3') were used. The *N* gene primers (*N*-F: 5’-GGG GAA CTTC TCC TGC TAG AAT-3’, *N*-R: 5’- CAG ACA TTT TGC TCT CAA GCT G-3’) and probe (*N*-P 5’-FAM-TTG CTG CTG CTT GAC AGA TT-TAMRA-3’) were used to confirm the RNA samples with cycle threshold values between 37 and 40. Viral genome copy numbers were calculated based on a standard curve generated from the *in vitro* transcribed RNAs that contained the PCR amplicon. All the primers and probes were designed according to the reference sequences of the SARS-CoV-2 genome (GISAID, <https://www.gisaid.org/>, accession number: EPI-ISL-402124)(1).

**Isolation of SARS-CoV-2 from ICU aerosols and masks**

Vero-E6 cells (ATCC CRL-1586) were cultured in Dulbecco’s minimal essential medium (DMEM) supplemented with 10% fetal bovine serum (Thermo Fisher Scientific, Thornton, Australia) and 100 units/mL penicillin and 1 mg/mL streptomycin (Invitrogen, NY, USA) in a humidified 37°C incubator with 5% CO_2_. Three hundred μl of the collected solutions from the air samples or patients’ masks were directly inoculated into the cells. For viral extraction of the masks, 4 cm^2^ sections were cut from near where the persons’ noses and mouths had been, and those aliquots were then soaked in 300 μL DMEM. At 2 h post-inoculation, the cell media were refreshed. The inoculated cells were passaged for three rounds every 3 days. The culture media was collected for viral RNA quantification by qRT-PCR.

**Fig. S1** Locations where samples were collected in and around the Wuhan Jinyintan hospital. (A) Sampling locations in the inpatient building. Red and green circles show the locations where the indoor aerosol samples were collected near patients with or without COVID-19, respectively. Blue circles show the locations where the outdoor aerosol samples were collected. The squares and triangles mark the locations where the mask samples and solid surface swabs were collected. Resting areas included a dressing room, nurse station, doctor’s office, resting rooms, and hallway. Corridors included a staff corridor, staff elevator, and corridor close to a computed tomography (CT) room. (B) Locations where samples were collected in the outpatient building. The outdoor samples were collected 10-m away from the buildings as indicated.


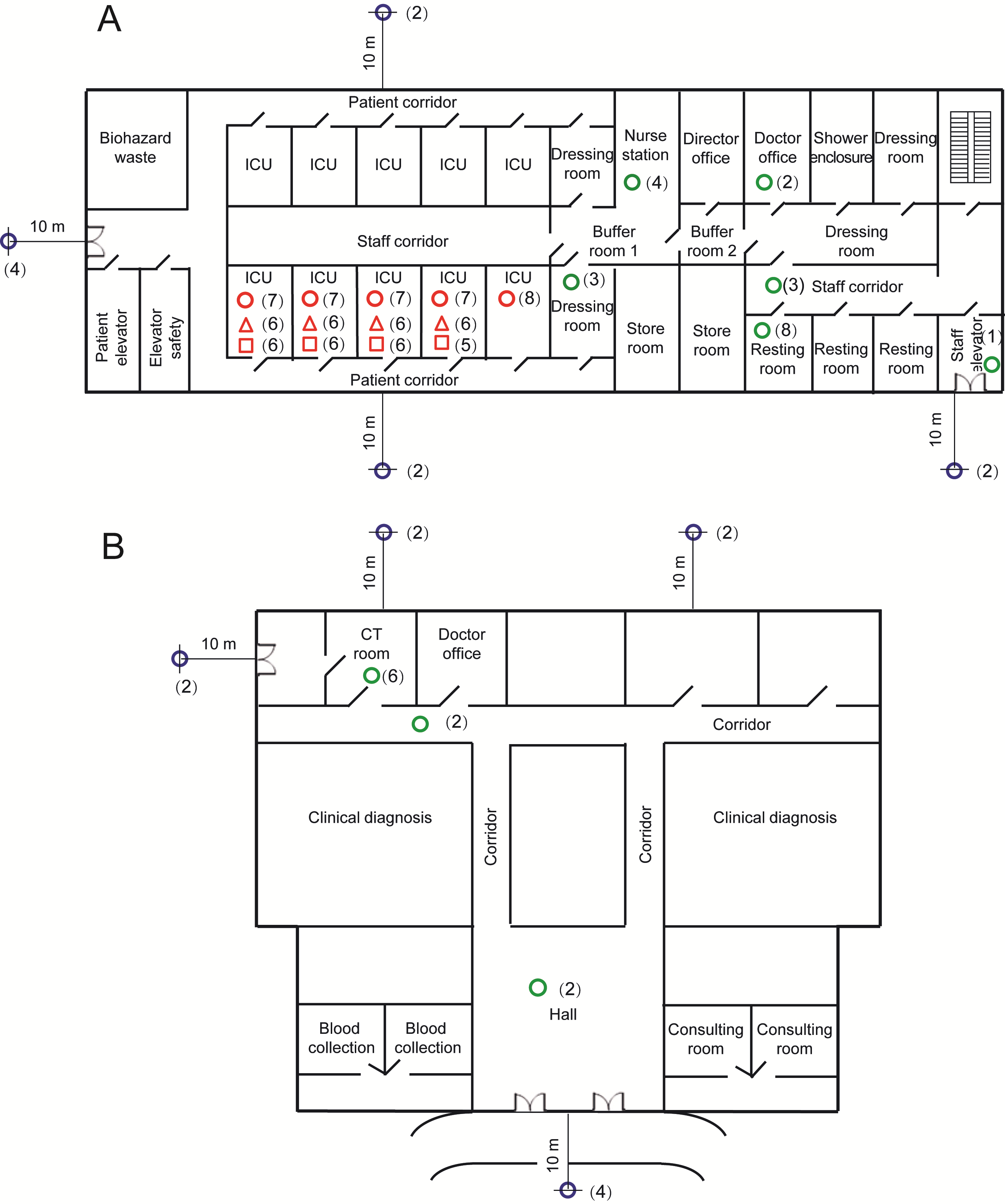


**Figure S2. Detection of SARS-CoV-2 in the masks and filters of respirators in Wuhan hospitals, China**

The masks that were collected from patients and healthy staff in the P3 laboratory, and respirator filters from the staff were tested for SARS-CoV-2 RNA by qRT-PCR.

A. The numbers of qRT-PCR positive and negative samples are shown as filled and open bars, respectively; the dashed line represents the qRT-PCR positive rates for the aerosol samples;

B. Copy numbers of SARS-CoV-2 RNA in the aerosols. The copy numbers were calculated as described in the legend of Figure. 1.

**Figure S3. Detection of SARS-CoV-2 on solid surfaces in Wuhan hospitals.**

Twenty four swabs from solid surfaces in intensive care units were collected and washed with phosphate buffered saline. The wash buffer was mixed with TRIzol Reagent to isolate RNA and analyzed by qRT-PCR.

Table S1. Specifications of sampling sites

| **Category** | **Site** | **Indoors or outdoors** | **Area size** | **Inpatient number/ symptom** | **Sampling date in 2020 (number)** |
| --- | --- | --- | --- | --- | --- |
| **Closed Environments in Hospitals** | | | | | |
| Union Hospital | Intensive care unit | Indoor | Na^1^ | 20/severe | 2/16 (2) |
|  | Resting areas | Indoor | na | --- | 2/16 (5) |
|  | Corridor | Indoor | na | --- | 2/16 (4) |
| Jinyintan Hospital | Intensive care unit | Indoor | 30 m^2^ | 2/severe | 2/16 (10) 2/19 (6) 2/21 (2) 2/24 (2) 3/5 (4) 3/8 (4) 3/12 (8) |
|  | Computerized tomography room | Indoor | 30 m^2^ | 1/confirmed | 2/17 (2) 2/24 (4) |
|  | Nurse station (resting area) | Indoor | na | --- | 2/16 (4) 2/25 (1) |
|  | Doctor’s office (resting area) | Indoor | na | --- | 2/16 (1) 2/25 (1) |
|  | Resting area | Indoor | na | --- | 2/16 (4) 2/17 (6) |
|  | Corridor | Indoor | na | --- | 2/16 (2) 2/17 (4) |
| Mobile Cabin Hospital | Hall | Indoor | > 500 m^2^ | > 200/mild | 3/2 (2) 3/9 (2) |
|  | Corridor |  | na | --- | 3/9 (1) |
| **Open Environments outside Hospitals** | | | | | |
| Jinyintan Hospital | Outpatient building | Outdoor |  | --- | 2/17 (4) 2/25 (2) 3/5 (2) 3/8 (2) |
|  | Inpatient building | Outdoor |  | --- | 2/25 (2) 3/5 (2) 3/8 (2) 3/14 (4) |
| Medical Observation Hotel | Hanting Hotel | Outdoor |  | --- | 2/27 (2) |
|  | Singer Rose Hotel | Outdoor |  | --- | 3/1 (2) |
| Residential Community | Keyuan West Community | Outdoor |  | --- | 2/26 (2) |
|  | Zhangjiawan Community | Outdoor |  | --- | 2/27 (2) |
|  | Jiyuqiao Community | Outdoor |  | --- | 3/1 (2) |
| Open Public Environment | Wuhan Institute of Virology | Outdoor |  | --- | 2/16 (2) 2/28 (4) |
|  | Eastlake Greenway | Outdoor |  | --- | 2/26 (2) |
|  | Botany Garden | Outdoor |  | --- | 2/27 (2) |
|  | East Lake | Outdoor |  | --- | 2/27 (2) |
| **Surface Samples from Intensive Care Unit** | | | | | |
| Jinyintan Hospital Intensive care unit | Cabinet | Indoor | 30 m^2^ | 2/severe | 2/21 (2) 3/5 (4) |
|  | Patient Monitor | Indoor | 30 m^2^ | 2/severe | 2/21 (2) 3/5 (4) |
|  | Door Handle | Indoor | 30 m^2^ | 2/severe | 2/21 (2) 3/5 (4) |
|  | Bed Rail | Indoor | 30 m^2^ | 2/severe | 2/21 (2) 3/5 (4) |
| **Mask Samples from Intensive Care Unit** | | | | | |
| Jinyintan Hospital | Mask | Indoor | 30 m^2^ | Mild | 3/8 (9) |
|  | Mask | Indoor | 30 m^2^ | Severe | 2/21 (2) 3/8 (6) |
|  | Mask | Indoor | 30 m^2^ | Critical | 3/8 (6) |
| **Mask and Respirator Samples from P3 Laboratory (Negative Pressure)** | | | | | |
| P 3 Laboratory in Wuhan Institute of Virology | Mask | Indoor | 22.5 m | Normal | 5/7 (40) |
|  | Respirator | Indoor | 22.5 m^2^ | Normal | 5/7 (9) |

^1^na stands for information not available
